# Impact of COVID-19 pandemic on the PREDIMED-Plus randomized clinical trial: Effects on the interventions, participants follow-up, and adiposity

**DOI:** 10.3389/fnut.2022.1098269

**Published:** 2023-01-12

**Authors:** Indira Paz-Graniel, Montserrat Fitó, Emilio Ros, Pilar Buil-Cosiales, Dolores Corella, Nancy Babio, J. Alfredo Martínez, Ángel M. Alonso-Gómez, Julia Wärnberg, Jesús Vioque, Dora Romaguera, José López-Miranda, Ramon Estruch, Francisco J. Tinahones, José Lapetra, Lluis Serra-Majem, Aurora Bueno-Cavanillas, Josep A. Tur, Vicente Martín-Sánchez, Xavier Pintó, José Juan Gaforio, Pilar Matía-Martín, Josep Vidal, Clotilde Vázquez, Lidia Daimiel, Jesus F. García-Gavilán, Estefanía Toledo, Stephanie K. Nishi, Jose V. Sorlí, Olga Castañer, Antonio García-Ríos, Manoli García de la Hera, Francisco Javier Barón-López, Miguel Ruiz-Canela, Marga Morey, Rosa Casas, Eva María Garrido-Garrido, Lucas Tojal-Sierra, José C. Fernández-García, Zenaida Vázquez-Ruiz, Rebeca Fernández-Carrión, Albert Goday, Patricia J. Peña-Orihuela, Laura Compañ-Gabucio, Helmut Schröder, Miguel A. Martínez-Gonzalez, Jordi Salas-Salvadó

**Affiliations:** ^1^Consorcio CIBER, Fisiopatología de la Obesidad y Nutrición (CIBERObn), Institute of Health Carlos III, Madrid, Spain; ^2^Universitat Rovira i Virgili, Departament de Bioquímica i Biotecnologia, Unitat de Nutrició, Reus, Spain; ^3^Institut d’Investigació Sanitària Pere Virgili (IISPV), Reus, Spain; ^4^Unit of Cardiovascular Risk and Nutrition, Institut Hospital del Mar de Investigaciones Médicas Municipal d‘Investigació Médica (IMIM), Barcelona, Spain; ^5^Lipid Clinic, Department of Endocrinology and Nutrition, Institut d’Investigacions Biomèdiques August Pi Sunyer (IDIBAPS), Hospital Clínic, Barcelona, Spain; ^6^Primary Health Care, IdiSNA, Servicio Navarro de Salud, Pamplona, Spain; ^7^School of Medicine, Department of Preventive Medicine and Public Health, University of Valencia, Valencia, Spain; ^8^Department of Nutrition, Food Sciences, and Physiology, Center for Nutrition Research, University of Navarra, Pamplona, Spain; ^9^Precision Nutrition and Cardiometabolic Health Program, IMDEA Food, CEI UAM + CSIC, Madrid, Spain; ^10^Bioaraba Health Research Institute, Osakidetza Basque Health Service, Araba University Hospital, University of the Basque Country UPV/EHU, Vitoria-Gasteiz, Spain; ^11^EpiPHAAN Research Group, School of Health Sciences, Instituto de Investigación Biomédica en Málaga (IBIMA), University of Málaga, Málaga, Spain; ^12^Centro de Investigación Biomédica en Red de Epidemiología y Salud Pública (CIBERESP), Madrid, España; ^13^Instituto de Investigación Sanitaria y Biomédica de Alicante, Universidad Miguel Hernández (ISABIAL-UMH), Alicante, Spain; ^14^Health Research Institute of the Balearic Islands (IdISBa), Palma de Mallorca, Spain; ^15^Department of Internal Medicine, Maimonides Biomedical Research Institute of Cordoba (IMIBIC), Reina Sofia University Hospital, University of Córdoba, Córdoba, Spain; ^16^Department of Internal Medicine, Institut d’Investigacions Biomèdiques August Pi Sunyer (IDIBAPS), Hospital Clinic, University of Barcelona, Barcelona, Spain; ^17^Department of Endocrinology, Instituto de Investigación Biomédica de Málaga (IBIMA), Virgen de la Victoria Hospital, University of Málaga, Málaga, Spain; ^18^Research Unit, Department of Family Medicine, Distrito Sanitario Atención Primaria Seville, Seville, Spain; ^19^Research Institute of Biomedical and Health Sciences (IUIBS), University of Las Palmas de Gran Canaria and Centro Hospitalario Universitario Insular Materno Infantil (CHUIMI), Canarian Health Service, Las Palmas, Spain; ^20^Department of Preventive Medicine and Public Health, University of Granada, Granada, Spain; ^21^Research Group on Community Nutrition and Oxidative Stress, University of Balearic Islands, Palma de Mallorca, Spain; ^22^Institute of Biomedicine (IBIOMED), University of León, León, Spain; ^23^Lipids and Vascular Risk Unit, Internal Medicine, Hospital Universitario de Bellvitge-Idibell, Hospitalet de Llobregat, Universitat de Barcelona, Barcelona, Spain; ^24^Departamento de Ciencias de la Salud, Instituto Universitario de Investigación en Olivar y Aceites de Oliva, Universidad de Jaén, Jaén, Spain; ^25^Department of Endocrinology and Nutrition, Instituto de Investigación Sanitaria Hospital Clínico San Carlos (IdISSC), Universidad Complutense, Madrid, Spain; ^26^Department of Endocrinology, Institut d’Investigacions Biomédiques August Pi Sunyer (IDIBAPS), Hospital Clinic, University of Barcelona, Barcelona, Spain; ^27^Department of Endocrinology and Nutrition, Hospital Fundación Jimenez Díaz, Instituto de Investigaciones Biomédicas IISFJD, University Autonoma, Madrid, Spain; ^28^Nutritional Control of the Epigenome Group, Precision Nutrition and Obesity Program, IMDEA Food, CEI UAM + CSIC, Madrid, Spain; ^29^Department of Preventive Medicine and Public Health, IDISNA, University of Navarra, Pamplona, Spain; ^30^Toronto 3D (Diet, Digestive Tract and Disease) Knowledge Synthesis and Clinical Trials Unit, Toronto, ON, Canada; ^31^Clinical Nutrition and Risk Factor Modification Centre, St. Michael’s Hospital, Unity Health Toronto, ON, Canada; ^32^Primary Care Center Zaidín-Center, Andalusian Health Service, Granada, Spain; ^33^Department of Nutrition, Harvard T.H. Chan School of Public Health, Boston, MA, United States

**Keywords:** COVID-19, PREDIMED-Plus, lockdown, clinical trial, weight-loss, Mediterraean diet

## Abstract

**Background:**

The COVID-19 pandemic has affected the implementation of most ongoing clinical trials worldwide including the PREDIMED-Plus study. The PREDIMED-Plus is an ongoing, multicenter, controlled intervention trial, aimed at weight-loss and cardiovascular disease prevention, in which participants were randomized (1:1 ratio) to an intervention group (energy-reduced Mediterranean diet, promotion of physical activity, and behavioral support) or to a control group (Mediterranean diet with usual care advice). When the pandemic began, the trial was in the midst of the planned intervention. The objective of this report was to examine the effects of the pandemic on the delivery of the intervention and to describe the strategies established to mitigate the possible adverse effects of the pandemic lockdown on data collection and adiposity.

**Methods:**

We assessed the integrity of the PREDIMED-Plus trial during 5 identified periods of the COVID-19 pandemic determined according to restrictions dictated by the Spanish government authorities. A standardized questionnaire was delivered to each of the 23 PREDIMED-Plus recruiting centers to collected data regarding the trial integrity. The effect of the restrictions on intervention components (diet, physical activity) was evaluated with data obtained in the three identified lockdown phases: pre lockdown, lockdown proper, and post lockdown.

**Results:**

During the lockdown (March/2020-June/2021), 4,612 participants (48% women, mean age 65y) attended pre-specified yearly follow-up visits to receive lifestyle recommendations and obtain adiposity measures. The overall mean (SD) of the proportions reported by each center showed that 40.4% (25.4) participants had in-person visits, 39.8% (18.2) participants were contacted by telephone and 35% (26.3) by electronic means. Participants’ follow-up and data collection rates increased across lockdown periods (from ≈10% at onset to ≈80% at the end). Compared to pre-lockdown, waist circumference increased during (0.75 cm [95% CI: 0.60–0.91]) and after (0.72 cm [95% CI: 0.56–0.89]) lockdown. Body weight did not change during lockdown (0.01 kg [95% CI: –0.10 to 0.13) and decreased after lockdown (-0.17 kg [95% CI: –0.30 to –0.04]).

**Conclusion:**

Mitigating strategies to enforce the intervention and patient’s follow-up during lockdown have been successful in preserving the integrity of the trial and ensuring its continuation, with minor effects on adiposity.

**Clinical trial registration:**

https://doi.org/10.1186/ISRCTN89898870, identifier ISRCTN89898870.

## 1. Introduction

In 2020, in an attempt to control Coronavirus disease 2019 (COVID-19) outbreaks, governments worldwide issued and enforced orders for social restrictions and lockdowns that had the potential to substantially affect the integrity of ongoing clinical trials. Since then, an important number of trials have been withdrawn or suspended and, in those in which it was decided to continue the trial, the pandemic particularly affected participant enrollment, the originally planned interventions, follow-up and data collection, and in some cases even outcome assessment (1). To mitigate potential consequences to participant safety and keep pace with the regulatory requirements established by authorities, clinical trial procedures often required amendments (2–4). In this regard, concerns regarding remote research and clinical trial integrity during and after the COVID-19 pandemic, especially regarding behavioral interventions, were reported early in 2020 (5). It was suggested that protocol modifications of ongoing clinical trials can potentially introduce biases, casting doubt on the trials’ validity and final conclusions, and therefore potential remote adaptations should be considered only after thorough reflection of the impact (5). In some cases, new statistical approaches may be necessary for the correct interpretation of trial results. Guidelines for reporting trial protocols and completed trials modified due to the COVID-19 pandemic and other extenuating circumstances have been established by the CONSERVE 2021 Statement Group and the WHO, which aim to provide guidance to improve the reporting of trials in which protocols had to be modified (1, 6). The PREDIMED-Plus is an ongoing large multicenter clinical trial conducted in Spain aiming to assess the effect of a lifestyle intervention on the primary prevention of cardiovascular disease (CVD) and mortality. At the time the COVID-19 pandemic began (March 2020 in Spain), recruitment was completed but the intervention was ongoing for most participants, thus several strategies had to be established to continue with the intervention and data collection remotely.

In this report, we aimed to describe how the COVID-19 pandemic affected the intervention delivery and the follow-up of participants enrolled in the PREDIMED-Plus trial. Mitigation strategies to minimize the effect of the lockdown in the different study sites are also delineated. Finally, we report the effect of the lockdown on the study outcomes of body weight and waist circumference.

## 2. Materials and methods

### 2.1. Study design and participants

The PREDIMED-Plus study is an ongoing 8-year (6 years of active intervention plus 2 years of follow-up without intervention), multicenter, parallel-group, clinical trial conducted in 23 Spanish centers aiming to evaluate the effect of a lifestyle intervention focused on weight loss via an energy-restricted Mediterranean Diet (erMedDiet), promotion of physical activity (PA), and behavioral support (intervention group) on CVD events and mortality compared to usual care advice promoting an *ad libitum* MedDiet (control group) in individuals with overweight/obesity and metabolic syndrome (MetS). PREDIMED-Plus participants (*n* = 6,874; aged 55–75 years) are men and women with overweight/obesity at baseline, free of CVD, and satisfying at least three criteria for the MetS (7), who at baseline were randomized in a 1:1 ratio to one of two intervention arms. To maintain the integrity of the trial the intervention and control groups will remain blinded and will be referred to as “Group A” and “Group B” without unblinding their true designation. The trial design and inclusion and exclusion criteria are detailed elsewhere (8). The study protocol can be accessed at www.predimedplus.com, and was registered on the ISRCTN registry (ISRCTN89898870). Both the protocol and procedures were implemented following the ethical standards of the Declaration of Helsinki and approved by the institutional ethics review boards of each study center. All participants provided written informed consent. At the time of writing this report, the intended recruitment (*n* = 6,874 participants) has been completed, participants had achieved the maximum weight-loss mean (achieved during the first intervention year) and were in the midst (a median [IQR] of 48.5 [43.8–58)] months of follow-up)of the planned intervention (receiving recommendations for both weight-loss and its long-term maintenance). The results of the pilot study concerning the effect of changes in body weight on CVD risk factors were published previously (9).

### 2.2. Lifestyle intervention and follow-up

Briefly, according to the PREDIMED-Plus study protocol, every 3 months, participants allocated to the active intervention group were to attend face-to-face visits with trained staff to receive intensive education to follow an erMedDiet, together with PA promotion and behavioral support aimed at achieving and maintaining weight loss. Additionally, participants were invited to participate in monthly group sessions providing dietary and PA counseling (12 sessions per year). Participants in the control group were to attend yearly face-to-face individual visits to receive recommendations on an *ad libitum* MedDiet, along with general lifestyle counseling, without specific advice related to PA or weight loss. In addition, every 6 months participants in the control group were to attend nutritional educational group sessions (2 sessions per year). The usual contact frequency (albeit not necessarily via face-to-face visits) between study personnel (dietitians and nurses) and participants was maintained during the lockdown phase. All participants received free extra-virgin olive oil (1 L/month) to reinforce their adherence to the MedDiet.

### 2.3. Anthropometric and lifestyle assessment

At baseline and yearly, all participants provided information on sociodemographic characteristics, lifestyle, PA, MedDiet adherence, among others. Anthropometric variables (height, weight, and waist circumference) were measured according to the study protocol. Body mass index (BMI) was calculated by dividing the weight (kg) by the height squared (m^2^). Adherence to an erMedDiet was assessed using a validated 17-item questionnaire in which the score ranged from 0 to 17, with 0 meaning current null adherence and 17 meaning maximum adherence (10). Leisure-time PA performed during a conventional month was assessed using the validated REGICOR questionnaire (11), which collected information about the type of activity, frequency (number of days) and duration (min/day). Sedentary time was evaluated on weekdays and weekends using the validated Nurse’s Health Study questionnaire for sedentary behaviors (12), which contained questions about the average daily time spent watching TV, using the computer, or sitting (at work, leisure time, or while traveling) during the last year. Possible responses included 12 categories ranging from 0 to ≥9 h/day of sitting time for the corresponding activity.

### 2.4. Sanitary restrictions in Spain due to the COVID-19 pandemic and changes established in the delivery of interventions during the lockdown

To slow disease transmission, a shelter-in-place order was enforced in the country by the Spanish government from March 14th to June 21st, 2020 (13). Following this, a transition plan was established to shift from severe lockdown to less restrictive conditions (14, 15). The National Health System in Spain is based on the coordination and integration between the state administration and 17 autonomous communities health services (16). As a consequence, during the post-lockdown phase (once the shelter-in-place order was raised), the severity of restrictions concerning mobility and social interactions varied in different Spanish regions according to both local COVID-19 prevalence/incidence and pressures on the health system.

As the PREDIMED-Plus trial intervention and data collection originally relied on in-person visits and group sessions, restrictions directly affected the day-to-day fieldwork of the trial. In March 2020, the trial was in the midst of the planned intervention, and to prematurely stop was not a sensible option given the study’s scientific value and allocated resources. Therefore, strategies were developed to continue the individual intervention and data collection. These strategies aimed to provide alternative means of study conduct, whether regulatory requirements prohibited or discouraged in-person visits or for when participants were reluctant to *attend study visits* in medical facilities for fear of COVID-19 contagion. All strategies established to continue the interventions and participant follow-up were approved by the PREDIMED-Plus Steering Committee.

During the lockdown, in response to the initial COVID-19 surge, the intervention was done remotely, with all necessary resources in order to do this remote conduct of the trial (including mobile phones and laptops) were provided to PREDIMED-Plus personnel. Study participants were contacted by telephone to receive nutritional and PA advice, and also to collect as much information as possible on lifestyle, dietary intake, and health status by using the same validated tools and procedure as in pre-lockdown conditions. In addition, digital material (e.g., MedDiet recommendations, recipes, at home PA promoting videos, etc.) were designed and sent by electronic means (text messaging, email, social networks) or uploaded to the participant accessible PREDIMED-Plus website to promote adherence to the interventions. For willing participants who had access to electronic devices with internet service, group sessions to continue nutrition intervention and PA promotion counseling were performed remotely by video calls.

Concerning anthropometric data, body weight was preferably measured in face-to-face interviews, but when in-person visits could not be performed this variable was self-reported or collected from clinical records (usual check-up visit with a medical doctor). Waist-circumference was measured by trained study staff at in-person study visits. During the initial complete lockdown, we did not collect biological samples, accelerometer data, or assess body composition (percentage of body fat, percentage of muscle mass, visceral adipose tissue measured by DXA).

After the initial lockdown period, when restrictions allowed, in-person visits were resumed. At the time this article was written (2021 last trimester), in-person visits were permitted across Spain. To ensure the safety and well-being of study participants and staff, standard operating procedures for site disinfection were developed and are enforced. Since the resumption of in-person visits, additional precautions have been taken (such as recommendations not to attend the study site if symptoms suspicious of COVID-19 infection are present and to report to the investigators any positive COVID-19 test results before coming to their study visit). Most group sessions continue to be conducted remotely by video calls due to local regulations.

### 2.5. Assessment of PREDIMED-Plus study integrity during the COVID-19 pandemic

Given the PREDIMED-Plus is a multicenter study, it was prone to be affected by region-specific health regulations. Therefore, between June and July 2021, a questionnaire designed to assess the general impact of the social and mobility restrictions enforced to slow the COVID-19 transmission on the PREDIMED-Plus intervention and data collection was sent to the principal investigators of each of the 23 recruitment centers.

**FIGURE 1:**
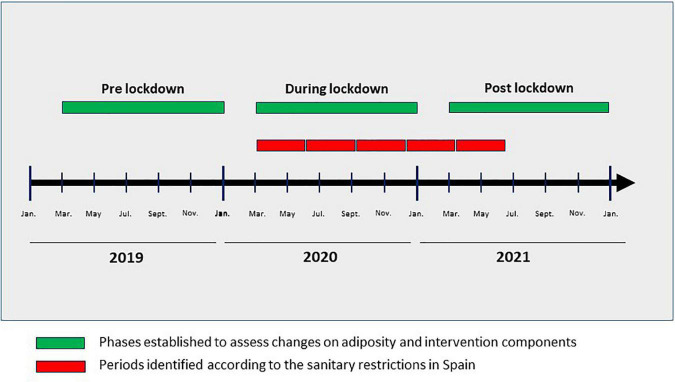
Lockdown periods assessed, and periods in which anthropometric measures, and components of the PREDIMED-Plus intervention were evaluated. Green colored boxes represent the three established lockdown phases to assess changes in adiposity and intervention components. Red colored boxes represent the five identified periods used to construct the questionnaire to assess the PREDIMED-Plus study integrity according to the prevalent sanitary restrictions in Spain.

The questionnaire was divided into five study periods (03-05/2020, 06-08/2020, 09-12/2020, 01-03/2021 and 04-06/2021) identified according to the timing of sanitary restrictions in Spain (Figure 1, represented by red colored bars). Furthermore, the questionnaire consisted of two parts. Part A included 8 questions related to the percentage of lockdown time and severity of restrictions applied. Responses could range from 0% “*There was no lockdown in that period*” to 100% “*There was total restriction throughout the period.*” Part B included 7 questions related to the proportions of the different methods of participant contact, data collection and intervention strategies used. Responses could range from 0% “*This method was not used*” to 100% “*This was the only method used.*” Responders (investigators involved in the day-to-day follow-up of participants) were asked to use only the percentages 25, 50 and 75% for intermediate situations (Supplementary material 1). The questionnaire was completed by the investigators of all PREDIMED-Plus centers (*n* = 23).

### 2.6. Effect of the pandemic lockdown on body weight and each intervention component

The present analysis was performed with data from participants who underwent the follow-up visits encompassed in the three identified phases concerning the COVID-19 pandemic situation in Spain (Figure 1, represented by green colored bars): pre-lockdown (March–December 2019), during lockdown (March–December 2020), and post-lockdown (March–December 2021). Participants without scheduled follow-up visits in any of the aforementioned phases or those for whom it was not possible to perform in-person visits due to the pandemic were excluded from the analyses concerning adiposity measurements, or changes in the individual components of the intervention (PA promotion, sedentary behavior, or erMedDiet adherence). The number of participants who should have had a face-to-face visit but did not have it during the lockdown phases was estimated.

### 2.7. Statistical analyses

For the present study, we used the PREDIMED-Plus database updated until December 2021. The proportion of participants receiving the intervention and data collection method during the COVID-19 pandemic are presented as mean (SD). Repeated-measures ANCOVA adjusted by sex and age was used to compare differences among the different lockdown phases in Spain on the main intervention variables. Paired Student *t*-tests were used for group comparisons (pre vs. during, pre vs. post, lockdown phases in Spain). Sensitivity analyses excluding participants with self-reported data were conducted for adiposity measurements. Stratified analyses by intervention group were also performed to assess changes in main intervention variables across lockdown periods. The proportion of participants who showed clinically meaningful changes [at least a 5% change in body weight, BMI, and waist circumference (17, 18)] during and post-lockdown phases was estimated. The data were analyzed using the Stata 14 software (StataCorp, College Station TX, USA), and statistical significance was set at a two-tailed p-value < 0.05.

## 3. Results

### 3.1. PREDIMED-Plus study integrity during the pandemic

**FIGURE 2:**
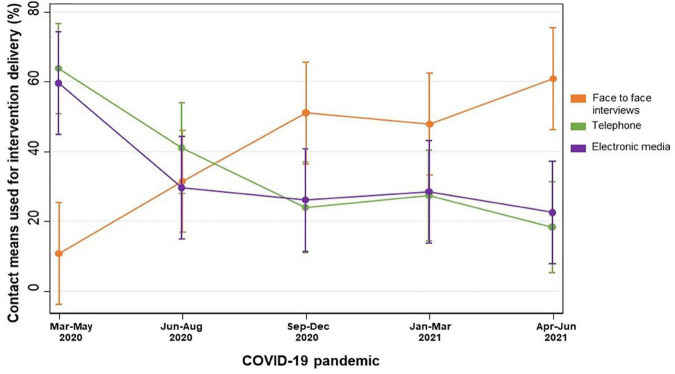
Individual intervention delivery methods applied in the PREDIMED-Plus trial during the COVID-19 pandemic in Spain. Means (SD) of the proportions of participants receiving the intervention during the COVID-19 pandemic.

The investigators from all 23 PREDIMED-Plus recruitment centers responded to the questionnaire assessing the impact of the COVID-19 pandemic on the integrity of the trial. Table 1 summarizes the mean lockdown rate in Spain from March 2020 to June 2021 and the reported percentages of participants receiving the intervention during the COVID-19 pandemic. The most severe lockdown rate (almost 100%) was reported from March to May 2020. During this period, individual nutritional and PA counseling, group sessions, and data collection through face-to-face interviews were scarcely used and participants were mainly contacted by telephone. Electronic means were also used to reinforce the intervention by developing and sharing digital materials with the participants. By the end of this assessment period (April-June 2021), face-to-face interviews had increased up to 60%; conversely, remote contacts by telephone or electronic means were reduced to 25%. Throughout the COVID-19-related study period, group sessions were mostly conducted by video calls rather than face-to-face (48.3 vs 9.8%). Figure 2 shows the individual intervention methods used during the lockdown periods in Spain throughout the pandemic. Compliance rates with PREDIMED-Plus study interventions by recruitment center and the mode of intervention delivery and data collection during the pandemic are displayed in Supplementary Table 1.

**TABLE 1:** PREDIMED-Plus study integrity during the coronavirus pandemic.

	COVID-19 (Mar-2020/Jun-2021)	Mar–May/20	Jun–Aug/20	Sep–Dec/20	Jan–Mar/21	Apr–Jun/21
Lockdown rate (%)		98.9 ± 5.2	32.6 ± 38.0	57.6 ± 32.4	37.4 ± 38.8	33.7 ± 35.0
**Performance by pandemic period, mean ± SD**						
**Individual nutritional and physical activity recommendations**						
In-person visit^I^	40.4 ± 25.4	10.9 ± 22.4	31.5 ± 38.6	51.1 ± 36.5	47.8 ± 37.6	60.9 ± 38.3
Telephone contact^I^	39.8 ± 18.2	67.4 ± 30.6	45.6 ± 35.1	29.3 ± 24.6	32.6 ± 30.6	23.9 ± 28.7
Contact via electronic means^I^	35.0 ± 26.3	59.8 ± 35.1	31.5 ± 32.2	28.3 ± 33.1	30.4 ± 32.8	25.0 ± 33.7
Group sessions						
– Face-to-face	9.8 ± 17.9	2.2 ± 7.2	6.5 ± 21.6	8.7 ± 25.7	13.0 ± 28.1	18.5 ± 33.9
– By videocall	48.3 ± 42.2	39.1 ± 47.0	42.4 ± 48.5	51.1 ± 49.1	55.4 ± 44.6	53.3 ± 46.7
Delivery of olive oil	69.3 ± 26.0	35.9 ± 46.4	70.7 ± 35.1	79.3 ± 27.8	80.4 ± 31.0	80.4 ± 31.0
Anthropometric measurements						
– Direct by study personnel	61.3 ± 20.3	11.9 ± 23.7	56.5 ± 36.3	77.2 ± 28.1	75.0 ± 32.0	85.9 ± 25.9
– Self-reported^ɣ^	34.2 ± 18.0	75.2 ± 33.2	39.1 ± 36.8	20.6 ± 26.8	22.8 ± 40.0	13.0 ± 23.7
Blood sample collection	61.1 ± 22.1	8.7 ± 23.4	65.2 ± 39.0	68.5 ± 36.3	78.3 ± 30.4	84.8 ± 23.5
Fecal and urine sample collection	60.2 ± 23.7	9.8 ± 25.8	64.1 ± 40.5	67.4 ± 35.7	77.2 ± 31.9	82.6 ± 26.6
Accelerometer data collection*	47.8 ± 30.9	11.3 ± 27.5	46.3 ± 40.0	52.5 ± 38.8	57.5 ± 45.2	71.3 ± 37.4

Data represent self-reported data from the 23 centers expressed as mean ± SD rates (%).

^I^Contact means could be used simultaneously.

^ɣ^Only used to collect data on body weight. *Accelerometer data collected in 20 of the 23 Predimed-Plus centers.

During the first and most severe lockdown period, in-person measurement of anthropometric variables and collection of biological samples was performed in <10% of participants (Table 1). Performance rates increased in subsequent COVID-19 restriction periods. The same pattern, but with lower proportions, occurred for accelerometer data collection. The percentage of participants with face-to-face anthropometric and accelerometer data measurements and biological sample collection by recruitment center during the lockdown are displayed in Supplementary Table 1.

### 3.2. Effect of the lockdown on body weight and each intervention component

**FIGURE 3:**
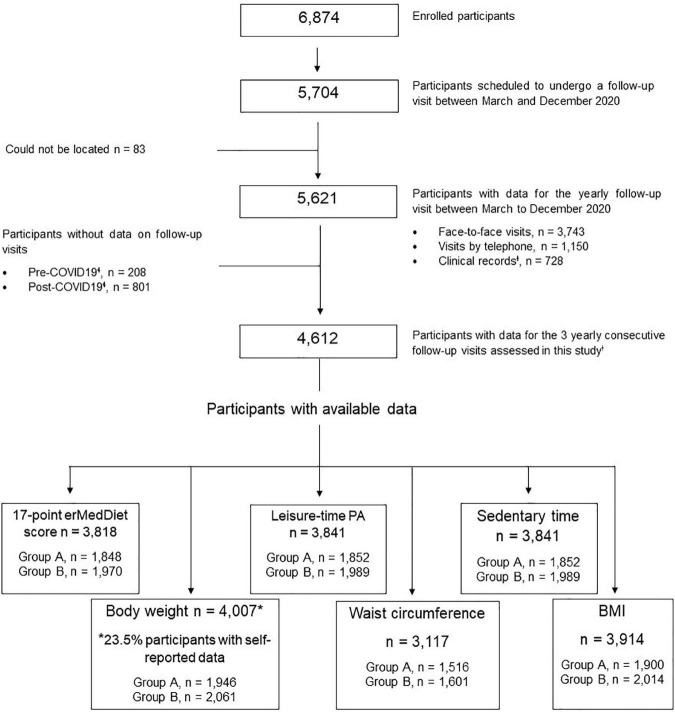
Flow diagram for study participants. P-Plus, PREDIMED-Plus study; BMI, body mass index; erMedDiet, energy-restricted Mediterranean diet; PA, physical activity; COVID-19, Coronavirus disease 2019. ^ɬ^Pre lockdown (from March to October 2019), during lockdown (from March to October 2020) and post-lockdown (from March to October 2021). ^Ɨ^To collect data on body weight.

During the lockdown phase (March to December 2020), according to the study protocol 5,704 participants should have attended a yearly follow-up visit. Of them, 65.6% had in-person visits, 32.9% were contacted by telephone or data were collected from clinical records, and 1.5% could not be reached. As not all participants completed all follow-up questionnaires, there were slightly different sample sizes for measurements of adiposity, 17-point erMedDiet score, leisure-time PA, and sedentary time (Figure 3).

The mean values of body weight, BMI, waist circumference, 17-point erMedDiet score, leisure-time PA, and sedentary time variables before, during, and after the COVID-19 pandemic are shown in Table 2. In the total cohort, no differences in BMI were observed; however, significant, albeit not clinically relevant, differences among lockdown periods were observed for body weight, waist circumference, the 17-point erMedDiet score, leisure-time PA, and sedentary time. After the exclusion of participants with self-reported data on body weight (23.5% of participants), similar differences were observed among the study periods (Table 2). A total of 13.0% of the participants exhibited a reduction ≥5% in body weight; while 13.7% of the individuals showed an increase>5% in waist circumference (Supplementary Table 2).

**TABLE 2:** Effect of the coronavirus pandemic (mean [95%CI] on intervention components and main outcome variables in the PREDIMED-Plus study (completers, *n* = 4,612).

	Pre lockdown (Mar–Dec/2019)	During lockdown (Mar–Dec/2020)	Post lockdown (Mar–Dec/2021)	*P-value*	During vs. Pre	Post vs. Pre
17-point erMedDiet score	11.8 (11.7–11.8)	11.8 (11.8–11.9)	11.6 (11.5–11.6)	<0.01	0.02 (–0.05 to 0.09)	–0.18 (–0.26 to –0.10)**
Leisure-time physical activity, METs.min/day	441.6 (434.0–449.2)	439.0 (431.4–446.7)	413.7 (406.1–421.3)	<0.01	–2.60 (–13.2 to 8.00)	–27.9 (–38.8 to –17.1)**
–Light	134.8 (131.1–138.5)	135.5 (131.8–139.2)	139.4 (135.7–143.1)	0.17	0.67 (–4.4 to 5.7)	4.6 (–0.9 to 10.1)
–Moderate	165.0 (159.7–170.3)	177.1 (171.9–182.4)	161.6 (156.7–166.9)	<0.01	12.1 (5.0–19.3)**	–3.7 (–10.9 to 4.2)
–Intense	141.8 (136.6–147.1)	126.4 (121.2–131.7)	112.7 (107.5–117.9)	<0.01	–15.4 (–22.8 to –8.1)**	–29.2 (36.6 to –21.8)**
Sedentary time, h/day	5.6 (5.5–5.6)	5.7 (5.6–5.7)	5.7 (5.6–5.7)	0.01	0.08 (0.02–0.13)**	0.07 (0.01–0.13)*
Body weight, kg^a^	83.9 (83.9–84.0)	84.0 (83.9–84.0)	83.8 (83.7–83.9)	<0.01	0.01 (–0.10 to 0.13)	–0.17 (–0.30 to –0.04)*
Measured body weight, kg^b^	83.9 (83.8, 84.0)	84.1 (84.0, 84.2)	83.8 (83.7, 83.9)	<0.01	0.19 (0.05, 0.32)**	–0.15 (–0.29, –0.00)*
BMI, kg/m^2c^	31.7 (31.6–31.7)	31.7 (31.7–31.8)	31.7 (31.6–31.7)	0.23	0.04 (–0.01 to 0.08)	–0.00 (–0.06 to 0.05)
Waist circumference, cm^d^	105.3 (105.2–105.4)	106.0 (105.9–106.1)	105.9 (105.9–106.1)	<0.01	0.75 (0.60–0.91)**	0.72 (0.56–0.89)**

BMI, body mass index; COVID-19, Coronavirus disease 2019; erMedDiet, energy-restricted MedDiet; MET, metabolic equivaluent of task. Data is expressed as % (*n*), mean ± SD, or as mean (95% CI). Group comparisons (pre-COVID-19, during COVID-19 and post-COVID-19 lockdown phases in Spain) were performed using repeated-measures ANCOVA adjusted for sex and age (*P* < 0.05).

Group comparisons (pre vs. during and pre vs. post phases in Spain) by paired t-test (*P < 0.05; **P < 0.01).

^a^Includes measured and self-reported weight, *n* = 4,007.

^b^After exclusion of 943 participants with self-reported weight, *n* = 3,064.

^c^Includes measured and self-reported weight *n* = 3,914.

^d^Includes only measured waist circumference, *n* = 3,117.

In the total cohort, waist circumference and sedentary time increased during lockdown (0.75 cm [0.60; 0.91]) and (0.08 h/day [0.02; 0.13]), respectively). After lockdown, body weight, the 17-point erMedDiet score, and PA significantly decreased in comparison with the pre-lockdown and lockdown phases (–0.17 kg [–0.30; –0.04], –0.18 points [–0.26; –0.10], and –27.9 METs.min/day [–38.8; –17.1], respectively). Waist circumference remained increased during the post-lockdown phase.

The effect of the COVID-19 pandemic on the main intervention variables of the trial by intervention group are displayed in Table 3. In both intervention groups, differences were observed for waist circumference, 17-point erMedDiet score, and leisure-time PA among assessment phases. In Group A, body weight decreased after lockdown. In Group B, increases in sedentary time were recorded during and post lockdown.

**TABLE 3:** COVID-19 pandemic effect (mean [95% CI]) over the main intervention variables in the PREDIMED-Plus trial by intervention group.

	Group A				Group B			
	**Pre lockdown (Mar–Dec/2019)**	**During lockdown (Mar–Dec/2020**	**Post lockdown (Mar–Dec/2021)**	***P-value***	**Pre lockdown (Mar–Dec/2019)**	**During lockdown (Mar–Dec/2020**	**Post lockdown (Mar–Dec/2021)**	***P-value***
17-point erMedDiet score	*n* = 1,848				*n* = 1,970			
	12.9 (12.8–13.0)	12.8 (12.7–12.8)	12.6 (12.5–12.6)	<0.01	10.7 (10.7–10.8)	10.9 (10.8–11.0)	10.7 (10.6–10.8)	< 0.01
Leisure-time PA, METs/min/day	*n* = 1,852				*n* = 1,989			
	499.1 (488.0–510.1)	484.9 (473.9–496.0)	459.9 (459.9–471.0)	<0.01	388.2 (377.7–398.6)	396.3 (385.8–406.8)	370.6 (360.2–381.1)	<0.01
Sedentary time, h/day	*n* = 1,852				*n* = 1,989			
	5.5 (5.4–5.5)	5.5 (5.5–5.6)	5.5 (5.5–5.6)	0.83	5.7 (5.6–5.7)	5.8 (5.7–5.8)	5.8 (5.7–5.9)	0.01
Body weight, kg	*n* = 1,946				*n* = 2,061			
	82.7 (82.6–82.8)	82.7 (82.6–82.8)	82.5 (82.4–82.6)	0.02	85.1 (85.0–85.3)	85.2 (85.0–85.3)	85.0 (84.8–85.1)	0.09
BMI, kg/m^2^	*n* = 1,900				*n* = 2,014			
	31.1 (31.1–31.2)	31.2 (31.1–31.2)	31.1 (31.1–31.2)	0.39	32.2 (32.2–32.3)	32.3 (32.2–32.3)	32.2 (32.2–32.3)	0.50
Waist circumference, cm	*n* = 1,516				*n* = 1,601			
	103.5 (103.3–103.6)	104.1 (104.0–104.3)	104.2 (104.1–104.7)	<0.01	107.0 (106.8–107.1)	107.8 (107.6–108.0)	107.7 (107.5–107.8)	< 0.01

BMI, body mass index; COVID-19, Coronavirus disease 2019; erMedDiet, energy-restricted MedDiet; MET, metabolic equivalent of task; PA, physical activity. Data is presented as mean (95% CI). Group comparisons (pre-COVID-19, during COVID-19 and post-COVID-19 lockdown periods in Spain) were performed using repeated-measures ANCOVA adjust for sex and age (*P* < 0.05).

## 4. Discussion

The present work describes the effect of the COVID-19 pandemic lockdown and related social restrictions on the delivery of the PREDIMED-Plus interventions and participants’ follow-up. We also report the strategies applied to mitigate the influence of social and mobility restrictions on the conduct of the trial, and the effect of the lockdown on the main components of the trial. Despite the strict shelter-in-place orders enforced in Spain, the intervention was not interrupted at any point of the pandemic. This was possible thanks to the early implementation of remote contact strategies (telephone, social networks, videocall platforms), and likely due to the motivated nature and possibly feelings of empowerment expressed by the participants who had been enrolled in the trial for a median [IQR] of 48.5 [43.8–58)] months when the pandemic began. During pre-lockdown, lockdown proper, and post-lockdown phases we also observed significant, albeit clinically irrelevant, differences in body weight, waist circumference, leisure-time PA, sedentary time, and adherence to the erMedDiet, while no changes were reported for BMI.

### 4.1. COVID-19 pandemic lockdown and changes in delivery of the PREDIMED-Plus trial intervention

Since March 2020, uninterrupted measures against COVID-19 have been enforced in Spain. However, the degree of sanitary restrictions, lockdown, and curfews have varied over time across the Spanish territory. Differences in the severity of restrictions and in human and economic resources determined the methods and tools used at each center during the lockdown. These conditions have impacted PREDIMED-Plus fieldwork so that intervention delivery methods had to be modified, but the prompt response of study investigators enabled the trial to continue. Even though face-to-face fieldwork was substantially affected, individual nutritional and PA counseling was conducted in all recruitment centers through alternative approaches, and importantly the trial was not stopped at any point.

The use of technology for the remote delivery of individual counseling of nutrition and PA recommendations allowed study personnel to reach a large proportion of participants. In addition, the materials and tools designed to provide recommendations on diet and PA were focused on the home lockdown (avoiding unhealthy eating behaviors and snacking between meals, providing healthy recipes, PA video routines, etc.). The methods applied in the trial were in accordance with the advice subsequently delivered by the WHO and other research organizations suggesting the use of existing communication platforms (e.g., text messaging, email, and social media applications) to stay in touch with study participants during the pandemic (1, 6, 19). The use of electronic means, has been previously reported to be a suitable alternative for nutritional and PA promotion and data collection (1, 6, 19), in Spanish adult people (20), and the preferred strategy by older participants (2).

Biological samples and accelerometer variables were not collected during the initial lockdown period due to the restrictions and difficulties associated with participants being able to attend face-to-face visits, and the little information available regarding safety protocols for the collection, management, and storage of biological samples. Nevertheless, once the restrictions were eased and recapture protocols were established and approved in each center, the percentages of data collection showed a linear increase.

Educational group sessions were the most affected intervention component. First, due to the social and mobility restrictions that did not permit meetings, and second because older individuals tend to have little knowledge of how to use existing communication platforms. Up to July 2021, only a few centers had resumed in-place meetings, while most centers conducted group sessions via video calls, and only 4 centers reported not conducting any group sessions.

### 4.2. Effect of the COVID-19 pandemic on body weight and main components of the intervention

It is important to underline that PREDIMED-Plus is a trial in individuals with overweight/obesity in which the principal aim is weight loss and its long-term maintenance. When the confinement began, participants were in the phase of weight loss maintenance. In the present analysis, conducted with data from 67% of the total recruited participants, we observed no differences among lockdown phases regarding mean BMI. Yet, body weight, waist circumference, erMedDiet adherence, leisure-time PA, and sedentary time showed variations among lockdown periods. However, the slight increases in adiposity measurements observed during confinement were clinically irrelevant. Moreover, after lockdown, both anthropometric indicators decreased. Similar results were observed when participants with self-reported data on body weight were excluded from the analyses.

Results from previous surveys have reported increased body weight during COVID-19 lockdowns (21–23). Discrepancies regarding the degree of weight gain between our study and other reports might be due to the PREDIMED-Plus objective and design, which might have played an important role in the prevention of weight gain. Moreover, data from most of the aforementioned studies were self-reported and not measured by trained staff, as some participants were reluctant to attend in-person visits with study personnel, due to their high risk of COVID-19 complications and mortality because of potential underlying health conditions (24).

It has been reported that the pandemic and confinement affected eating behaviors (20). Interestingly, dietary changes during the pandemic were beneficial in our participants, as an increase in adherence to the intended intervention with erMedDiet was observed. This can be explained by: (a) the continuous advice received by the participants concerning healthy dietary behaviors, (b) the messages provided to them about the potential protective effect of a healthy diet and weight loss against COVID-19 complications, and, (c) the temporary closure of cafeterias, bars, and restaurants and prohibition of social encounters. These results are in line with those reported in a recent systematic review in which a worldwide moderate improvement in dietary habits was observed during lockdown (20); and with other surveys conducted in Mediterranean populations, which reported higher adherence to the MedDiet during COVID-19 confinement (25, 26). However, an increase in the consumption of certain “unhealthy” food groups (27) or alcohol (28), and an increase in the prevalence of eating behaviors such as snacking or overconsumption of ultra-processed foods, have also been reported (20, 29, 30).

Leisure-time PA showed a minimum decrease during the lockdown. This observation might be partly explained by a collateral effect of severe confinement in Spain. While during the first severe confinement trimester, outdoors sports were forbidden, during the transition phase, just after the initial lockdown, an increased number of people, more than usual, were observed on the streets performing PA. This observation is in line with previous results reporting an increase in PA during the confinement in two Italian cohorts (25). In addition, it should be noted that half of the PREDIMED-Plus participants are receiving a PA promotion program and received advice for increased at-home training. This might partially explain the differences observed with other studies that reported decreased PA and increased sedentary behaviors during the confinement (21, 31, 32). This potential explanation is supported by the results of sedentary time observed for the intervention “Group B,” whose participants showed a significantly higher sedentary time during and post-lockdown compared to the pre-lockdown phase. Even so, it should also be noted that after lockdown, we observed a decrease in leisure-time PA intensity. This might be partly explained by the reluctance of participants to perform group PA indoors or outdoors. As PA promotion is one of the trial’s main intervention components, PREDIMED-Plus staff acknowledge the need to further emphasize this lifestyle factor for future compliance.

The main strength of our study is its prospective design. The pre-lockdown data availability allowed for comparison of events during lockdown and post-lockdown periods, which provided the opportunity to assess the potential impact of confinement and the strategies applied to mitigate its consequences on our main intervention variables. Also, validated tools were used to assess the main components of the lifestyle intervention: adherence to erMedDiet, PA, and sedentary behaviors (10–12). Our study also has limitations. First, responses to the questionnaire used to assess the integrity of the PREDIMED-Plus trial during and after the COVID-19 pandemic were self-reported and the mean lockdown rate per period was estimated from reports of PREDIMED-Plus centers and does not represent real data from the communities or Spanish restrictions. Second, not all PREDIMED-Plus participants were included in this analysis. Data from participants who attended a follow-up visit in 2020 before confinement began were excluded (for better representativeness of the effect of the restrictions on the trial components), and this might have induced selection bias. Third, results from changes in sedentary time should be interpreted with caution as the validated Nurse’s Health Study questionnaire for sedentary behaviors reflects data from the last year, and might not reflect the acute lockdown effect. Finally, contact frequency between study staff and the intervention groups differs throughout the trial, and this could have promoted different magnitudes of effect in adiposity and intervention adherence between the two groups during the lockdown. As, in this analysis, we kept the intervention blinded to the researchers, conclusions by intervention arms should be done with caution.

Remote visits and intervention, have limitations, but when applied in the PREDIMED-Plus study proved to be feasible and largely effective to retain research participants and keep the trial ongoing in an unpredictable environment. At the time this manuscript was written, the majority of the primary outcome assessments related to weight loss and weight-loss maintenance (i.e., body weight, waist circumference, dietary adherence, and other lifestyle variables) have been successfully collected.

## 5. Conclusion

In the ongoing PREDIMED-Plus trial, the COVID-19 pandemic lockdown affected the delivery of interventions, the follow-up of participants, and data collection, without significantly affecting the study design or resulting in major changes to the protocol (except for group sessions). Due to the strategies implemented to address restrictions related to the pandemic lockdown, the trial was not stopped at any moment during the COVID-19 pandemic. The strategies established resulted in small effects on the different intervention components. Compared to the pre-lockdown period, participant waist circumference and measured total body weight slightly increased during the lockdown in both intervention groups.

## PREDIMED-Plus study investigators

*Steering Committee:*

J. Salas-Salvadó (Coordinator), M.A. Martínez-González, M. Fitó. E. Ros, FJ. Tinahones, D. Corella and R. Estruch.

*Executive Committee:*

J. Salas-Salvadó, M.A. Martínez-González, D. Corella, M. Fitó, J. Vioque, D. Romaguera, J.A Martínez, J. Wärnberg, J. Lopez-Miranda, R. Estruch, A. Bueno-Cavanillas, Á.M. Alonso-Gómez, J.A. Tur, FJ. Tinahones, L. Serra-Majem, V. Martin, J. Lapetra, C. Vázquez, X. Pinto, J. Vidal, L. Daimiel, M. Delgado-Rodríguez, M.A. Rubio and E. Ros.

*Dietary and Lifestyle Intervention Committee:*

J. Salas-Salvadó (chair), M.A. Martínez-González, M. Fitó and R. Estruch;

*Dietary Intervention*: J. Salas-Salvadó (chair), N. Babio, E. Ros, A. Sánchez-Tainta;

*Physical Exercise*: M. Fitó (chair), H. Schröder, A. Marcos, D. Corella, J. Warnberg;

*Behavioural support*: R. Estruch (chair), F. Fernández-Aranda, C. Botella and J. Salas-Salvadó.

*Clinical Event Ascertainment Committee:*

F. Arós (Chair), A. Alonso-Gómez, M. Villas-Roca, M. Aldamiz, O. García-Regata, L. Forga, A. García-Layana, A. González-Pinto, I. Zorrilla, M. Martínez-Kareaga, P. Seoane.

Chair: Dr. Fernando Arós

Cardiology: Dr. Angel Alonso-Gómez; Dr. Fernando Arós

Neurology: Dr. Mònica Villas-Roca

Internal Medicine: Dr. Mikel Aldamiz; Dr. Óscar García Regata

Endocrinology: Dr. Lluis Forga

Ophthalmology: Dr. Alfredo García-Layana

Psychiatry: Dr. Ana González Pinto; Dr. Iñaki Zorrilla

Oncology: Dr. Mireia Martínez; Dr. Patricia Seoane

*Support groups:*

C. Botella, F. Fernandez-Aranda, R. Lamuela, A. Marcos, M.P. Portillo, E. Ros, G. Sáez, F. Arós, E. Gómez-Gracia.

*Rovira i Virgili University, Department of Biochemistry and Biotechnology, Human Nutrition Unit, University Hospital of Sant Joan de Reus, Pere Virgili Institute for Health Research, Reus, Spain:* R. Pedret Llaberia, R. Gonzalez, R. Sagarra Álamo, F. París Palleja, J. Balsells, J.M. Roca, T. Basora Gallisa, J. Vizcaino, P. Llobet Alpizarte, C. Anguera Perpiñá, M. Llauradó Vernet, C. Caballero, M. Garcia Barco, M.D. Morán Martínez, J. García Rosselló, A. Del Pozo, C. Poblet Calaf, P. Arcelin Zabal, X. Floresví, M. Ciutat Benet, A. Palau Galindo, J.J. Cabré Vila, F. Dolz Andrés, M. Soler, M. Gracia Vidal, J. Vilalta J. Boj Casajuana, M. Ricard, F. Saiz, A. Isach, M. Sanchez Marin Martinez, E. Granado Font, C, Lucena Luque, C. Mestres Sola, N. Babio, N. Becerra-Tomás, J. Basora, I. Abellán Cano, JF García-Gavilán, V. Ruiz García, C. Gomez-Martinez, L. Lopez-Gonzalez, I. Paz-Graniel, L. Sánchez Niembro, A. Díaz-López, S. Manzanedo, A. Atzeni, C. Valle. M, Fernández de la Puente, T. Garcidueñas-Fimbres, S Nishi, J Ni, S De las Heras, S Rios, C Munne, N Khoury, M Pascual, S Segura.

*Department of Preventive Medicine and Public Health, University of Navarra-Navarra Institute for Health Research (IdiSNA), Pamplona, Spain:* M. Ruiz-Canela, E. Toledo, P. Buil-Cosiales, Z. Vázquez, C. Razquin, M. Bes-Rastrollo, A. Gea, A. Sanchez Tainta, B. SanJulian Aranguren, E. Goñi, L. Goñi, M.J. Cobo, A. Rico-Campa, F.J. Basterra Gortari, A. Garcia Arellano, J. Diez-Espino, O. Lecea-Juarez, J. Carlos Cenoz-Osinaga, I. Alvarez-Alvarez, M.C. Sayon-Orea, C.I. Fernandez-Lázaro, L. Ruiz-Estigarribia, J. Bartolome-Resano, A. Sola-Larraza, E. Lozano-Oloriz, B. Cano-Valles, S. Eguaras, E. Pascual Roquet-Jalmar, I. Galilea-Zabalza, H. Lancova, R. Ramallal, M.L. Garcia-Perez, V. Estremera-Urabayen, M.J. Ariz-Arnedo, C. Hijos-Larraz, C. Fernandez-Alfaro, B. Iñigo-Martinez, R. Villanueva Moreno, S. Martin-Almendros, L. Barandiaran-Bengoetxea, C. Fuertes-Goñi, A. Lezaun-Indurain, M.J. Guruchaga-Arcelus, O. Olmedo-Cruz, L. Escriche-Erviti, R. Ansorena-Ros, R. Sanmatin-Zabaleta, J. Apalategi-Lasa, J. Villanueva-Telleria, M.M. Hernández-Espinosa, L. Herrera-Valdez, L. Dorronsoro-Dorronsoro, Lourdes Echeverria-Lizarraga (†), J.A. Cabeza-Beunza, P. Fernández-Urretavizcaya, P. Gascó-García, C. Royo-Jimenez, J. Moran-Pí, F. Salazar-Fernández, F.J. Chasco-Ros, F. Cortés-Ugalde, J.J. Jurio-Burgui, P. Pascual-Pascual, A.I. Rodríguez-Ezpeleta, M. Esparza-Cáceres, C. Arroyo-Azpa, M. Rodríguez-Sanz de Galdeano, T. Forcen-Alonso, M. Armendariz-Marcotegui, A. Brugos-Larumbe, A. Arillo, B. López-Aisa, R. Sanmartin-Zabaleta, M. Hermoso de Mendoza-Macua.

*Department of Preventive Medicine, University of Valencia, University Jaume I, Conselleria de Sanitat de la Generalitat Valenciana, Valencia, Spain:* J.I. González, J.V. Sorlí, O. Portolés, R. Fernández-Carrión, C. Ortega-Azorín, R. Barragán, E.M. Asensio, O. Coltell, I. Giménez-Alba, C. Sáiz, R. Osma, E. Férriz, I. González-Monje, P. Guillém-Sáiz, F. Giménez-Fernández, E.C. Pascual, L. Quiles, P. Carrasco, A. Carratalá-Calvo, C. Valero-Barceló, C. Mir, S. Sánchez-Navarro, J. Navas, J.J. Tamarit, I. González-Gallego, L. Bort-Llorca, L. Pérez-Ollero, M. Giner-Valero, R. Monfort-Sáez, J. Nadal-Sayol, V. Pascual-Fuster, M. Martínez-Pérez, C. Riera, M.V. Belda, A. Medina, E. Miralles, M.J. Ramírez-Esplugues, M. Rojo-Furió, G. Mattingley, M.A. Delgado, M.A. Pages, Y. Riofrío, L. Abuomar, N. Blasco-Lafarga, R. Tosca, L. Lizán, A.M. Valcarce, M.D. Medina, S. de Valcárcel, N. Tormo, O. Felipe-Román, S. Lafuente, I.M. Romero-Martínez, F.A. Rodríguez-Lagos, J.V. Crespo, J.L. Llosa, L. González-García, R. Raga-Marí.

*Cardiovascular Risk and Nutrition Research Group, Endocrinology Service, Neurosciences Programme, Clinical Research Unit at the Hospital del Mar Medical Research Institute (IMIM), Barcelona. Medicine Departament, Universitat Autònoma de Barcelona, Barcelona, Spain*: M. Fitó, O. Castañer, M.A. Muñoz, M.D. Zomeño, A. Hernaéz, L. Torres, M. Quifer, R. Llimona, G Freixer, KA. Pérez-Vega, M. Farràs, R. Elosua, J. Vila, I. Subirana, S. Pérez, A. Goday, J.J. Chillaron Jordan, J.A. Flores Lerroux, D. Benaiges Boix, G. Llauradó, M. Farré, E. Menoyo, A. Aldea-Perona, M. Pérez-Otero, D. Muñoz-Aguayo, S. Gaixas, G. Blanchart, A. Sanllorente, M. Soria, J. Valussi, A. Cuenca, L. Forcano, A. Pastor, A. Boronat, S. Tello, M. Cabañero, L. Franco, H. Schröder, R. De la Torre, C. Medrano, J. Bayó, M.T. García, V. Robledo, P. Babi, E. Canals, N. Soldevila, L. Carrés, C. Roca, M.S. Comas, G. Gasulla, X. Herraiz, A. Martínez, E. Vinyoles, J.M. Verdú, M. Masague Aguade, E. Baltasar Massip, M. López Grau, M. Mengual, V. Moldon, M. Vila Vergaz, R. Cabanes Gómez, Ciurana, M. Gili Riu, A. Palomeras Vidal, F Peñas F, A Raya, M.A. Sebastian, M. Valls, J. Guerrero, M. Marne, E. Minguella, M. Montenegro, A. Sala, M.R. Senan, N. Talens, N. Vera.

*Nutritional Epidemiology Unit, Miguel Hernandez University, ISABIAL-FISABIO, Alicante, Spain:* J. Vioque, M. García-de-la-Hera, S. Gonzalez-Palacios, L. Torres-Collado, L. Compañ-Gabucio, A. Oncina-Canovas, L. Notario-Barandiaran, D. Orozco-Beltran, S. Pertusa Martínez, A. Asencio, I. Candela-García, J.M. Zazo, D. Vivancos Aparicio, N. Fernández-Brufal, J. Román Maciá, F. Ortiz Díaz, M. García Muñoz, C. Barceló, E. Martínez-García, M Damaj-Hamieh, M.C. Martínez Vergara, M.A. Sempere Pascual, S.J. Miralles Gisbert, A. González Botella, C.M. López García, R. Valls Enguix, N. Gómez Bellvert, V. Martínez Avilés, R. Lloret Macián, A. Pastor Morel, M. Mayor-Llorca, J.J. Ballester Baixauli, G. Notario García, M.A. Belmar-Bueno, E.P. Cases Pérez, C. Tercero Maciá, L.A. Mira Castejón, J. Torregrosa García, C. Pastor Polo, E. Puig Agulló, M.V. Hernándis Marsán, M.J. González Fajardo, I. Hervella Durantez, M.C. Latorre Use, A. Bernabé Casanova, F. Medina Ruzafa, E. Robledano, I. Vilanova Martínez, A. Molina Santiago.

*Hospital Son Espases (HUSE) and Institute for Health Research Illes Balears (IdISBa), Palma de Mallorca, Spain:* M. Fiol, M. Moñino, A. Colom, J. Konieczna, A. Chaplin, M. Morey, L. Prohens, A.M. Galmés-Panadés, E. Rayó, J. Llobera, J. Fernández-Palomeque, E. Fortuny, M. Noris, L. López, X. Rosselló, S. Munuera, F. Tomás, F. Fiol, A. Jover, J.M. Janer, C. Vallespir, I. Mattei, N. Feuerbach, M. del Mar Sureda, S. Vega, L. Quintana, A. Fiol, M. Amador, S. González, J. Coll, A. Moyá, T. Piqué Sistac, M.D. Sanmartín Fernández, M.C. Piña Valls, M.A. Llorente San Martín, J. Pou Bordoy.

*Department of Nutrition, Food Sciences, and Physiology, Center for Nutrition Research, University of Navarra, Pamplona, Spain:* I. Abete, I. Cantero, C. Cristobo, I. Ibero-Baraibar, M. Zulet, J. Ágreda-Peiró, M.D. Lezáun-Burgui, N. Goñi-Ruiz, R. Bartolomé-Resano, E. Cano-Cáceres, T. Elcarte-López, E. Echarte-Osacain, B. Pérez-Sanz, I. Blanco-Platero, A. Andueza- Azcárate, A. Gimeno-Aznar, E. Ursúa-Sesma, B. Ojeda-Bilbao, J. Martinez-Jarauta, L. Ugalde-Sarasa, B. Rípodas-Echarte, M.V. Güeto-Rubio, C. Napal-Lecumberri, MD Martínez-Mazo, E Arina-Vergara, A. Parra-Osés, F. Artal-Moneva, F. Bárcena-Amigo, F. Calle-Irastoza, J. Abad-Vicente, J.I. Armendáriz-Artola, P. Iñigo-Cibrian, J. Escribano-Jarauta, J. Ulibarri-delportillo, B. Churio-Beraza, Y. Monzón-Martínez, E. Madoz-Zubillaga, C. Arroniz.

*University of Málaga and Institute of Biomedical Research in Malaga (IBIMA), Málaga, Spain:* F.J. Barón-López, J.C. Fernández García, N. Pérez-Farinós, N. Moreno-Morales, M. del C. Rodríguez-Martínez, J. Pérez-López, J.C. Benavente-Marín, E. Crespo Oliva, E. Contreras Fernández, F.J. Carmona González, R. Carabaño Moral, S. Torres Moreno, M.V. Martín Ruíz, M. Alcalá Cornide, V. Fuentes Gómez.

*Lipids and Atherosclerosis Unit, Department of Internal Medicine, Maimonides Biomedical Research Institute of Cordoba (IMIBIC), Reina Sofia University Hospital, University of Córdoba, Cordoba, Spain:* J. López-Miranda, A. Garcia-Rios, J. Criado García, A.I. Jiménez Morales, A. Ortiz Morales, J.D. Torres Peña, F.J. Gómez Delgado, J.F. Alcalá, A. León Acuña, A.P. Arenas Larriva, F. Rodríguez Cantalejo, J. Caballero Villaraso, I. Nieto Eugenio, P. Coronado Carvajal, M.C del Campo Molina, P.J. Peña Orihuela, I. Perez Corral, G. Quintana Navarro.

*Department of Internal Medicine, Institut d’Investigacions Biomèdiques August Pi i Sunyer (IDIBAPS), Hospital Clínic, University of Barcelona, Barcelona, Spain:* R. Casas, M. Domenech, C. Viñas, S. Castro-Barquero, A.M. Ruiz-León, R. Losno, L. Tarés, A. Jordán, R. Soriano, M. Camafort, C. Sierra, E. Sacanella, A. Sala-Vila, J. M. Cots, I. Sarroca, M. García, N. Bermúdez, A. Pérez, I. Duaso, A. de la Arada, R. Hernández, C. Simón, M.A. de la Poza, I. Gil, M. Vila, C. Iglesias, N. Assens, M. Amatller, LL. Rams, T. Benet, G. Fernández, J. Teruel, A. Azorin, M. Cubells, D. López, J.M. Llovet, M.L. Gómez, P. Climente, L. de Paula, J. Soto, C. Carbonell, C. Llor, X. Abat, A. Cama, M. Fortuny, C. Domingo, A. I. Liberal, T. Martínez, E. Yañez, M. J. Nieto, A. Pérez, E. Lloret, C. Carrazoni, A. M. Belles, C. Olmos, M. Ramentol, M. J. Capell, R. Casas, I. Giner, A. Muñoz, R. Martín, E. Moron, A. Bonillo, G. Sánchez, C. Calbó, J. Pous, M. Massip, Y. García, M.C. Massagué, R. Ibañez, J. Llaona, T. Vidal, N. Vizcay, E. Segura, C. Galindo, M. Moreno, M. Caubet, J. Altirriba, G. Fluxà, P. Toribio, E. Torrent, J. J. Anton, A. Viaplana, G. Vieytes, N. Duch, A. Pereira, M. A. Moreno, A. Pérez, E. Sant, J. Gené, H. Calvillo, F. Pont, M. Puig, M. Casasayas, A. Garrich, E. Senar, A. Martínez, I. Boix, E. Sequeira, V. Aragunde, S. Riera, M. Salgado, M. Fuentes, E. Martín, A. Ubieto, F. Pallarés, C. Sala, A. Abilla, S. Moreno, E. Mayor, T. Colom, A. Gaspar, A. Gómez, L. Palacios, R. Garrigosa.

*Departament of Preventive Medicine and Public Health, University of Granada, Granada, Spain:* L. García Molina, B. Riquelme Gallego, N. Cano Ibañez, A. Maldonado Calvo, A. López Maldonado, E.M. Garrido, A. Baena Dominguez, F. García Jiménez, E. Thomas Carazo, A. Jesús Turnes González, F. González Jiménez, F. Padilla Ruiz, J. Machado Santiago, M.D. Martínez Bellón, A. Pueyos Sánchez, L. Arribas Mir, R. Rodríguez Tapioles, F. Dorador Atienza, L. Baena Camus, C. Osorio Martos, D. Rueda Lozano, M. López Alcázar, F. Ramos Díaz, M. Cruz Rosales Sierra, P. Alguacil Cubero, A. López Rodriguez, F. Guerrero García, J. Tormo Molina, F. Ruiz Rodríguez.

*Bioaraba Health Research Institute, Cardiovascular, Respiratory and Metabolic Area; Osakidetza Basque Health Service, Araba University Hospital; University of the Basque Country UPV/EHU, Vitoria-Gasteiz, Spain:* I. Salaverria, A. Alonso-Gómez, M.C. Belló, L. Tojal, L. Goicolea, C. Sorto, A Goikoetxea, A. Casi Casanellas, M.L. Arnal Otero, J. Ortueta Martínez De Arbulo, J. Vinagre Morgado, J. Romeo Ollora, J. Urraca, M.I. Sarriegui Carrera, F.J. Toribio, E. Magán, A. Rodríguez, S. Castro Madrid, M.T. Gómez Merino, M. Rodríguez Jiménez, M. Gutiérrez Jodra, B. López Alonso, J. Iturralde Iriso, C. Pascual Romero, A. Izquierdo De La Guerra.

*Research Group on Community Nutrition & Oxidative Stress, University of Balearic Islands, Palma de Mallorca, Spain:* E. Angullo-Martinez, E. Argelich, M.M. Bibiloni, P.A. Borràs, C. Bouzas, J.M. Gámez, S. García, C. Gómez, I. Llompart, D. Mateos, M, Monserrat-Mesquida, S. Montemayor, A. Pons, M. Quetglas-Llabrés, T. Ripoll, T. Rodríguez, M. Ródenas, A. Sureda, S. Tejada, L. Ugarriza.

*Virgen de la Victoria Hospital, University of Málaga, Málaga, Spain:* M.R. Bernal López, M. Macías González, J. Ruiz Nava, J.C. Fernández-García, A. Muñoz Garach, A. Vilches Pérez, A. González Banderas, A.V. Alarcón-Martín, M. García Ruiz de Mier, J. Alcaide Torres, A. Vargas Candela, M. León Fernández, R. Hernández Robles, S. Santamaría Fernández, J.M. Marín, A. Gómez-Pérez, M. Damas-Fuentes.

*University of Las Palmas de Gran Canaria, Las Palmas, Spain:* J. Álvarez-Pérez, E.M. Díaz Benítez, F. Díaz-Collado, A. Sánchez-Villegas, J. Pérez-Cabrera, L.T. Casañas-Quintana, R.B. García-Guerra, I. Bautista-Castaño, C. Ruano-Rodríguez, F. Sarmiento de la Fe, J.A. García-Pastor, B. Macías-Gutiérrez, I. Falcón-Sanabria, C. Simón-García, A.J. Santana-Santana, J.B. Álvarez-Álvarez, B.V. Díaz-González, J.M. Castillo Anzalas, R.E. Sosa-Also, J. Medina-Ponce.

*Biomedicine Institute (IBIOMED); University of León, and Primary Health Care Management of León (Sacyl), León, Spain: Biomedicine Institute (IBIOMED); University of León, and Primary Health Care Management of León (Sacyl), León, Spain:* S. Abajo Olea, L. Álvarez-Álvarez, M. Rubín García, A. Torres, P. Farias, N. Cubelos, A. Adlbi Sibai, M. Ajenjo, E. Carriedo Ule, M. Escobar Fernández, J.I. Ferradal García, J.P. Fernández Vázquez, C. González Quintana, F. González Rivero, M. Lavinia Popescu, J.I. López Gil, J. López de la Iglesia, A. Marcos Delgado, C. Merino Acevedo, S. Reguero Celada, M. Rodríguez Bul, E. Fernández Mielgo.

*Department of Family Medicine, Distrito Sanitario Atención Primaria Sevilla, Seville, Spain:* J.M. Santos-Lozano, L. Miró-Moriano, C. Domínguez-Espinaco, S. Vaquero-Díaz, F.J. García-Corte, A. Santos-Calonge, C. Toro-Cortés, N. Pelegrina-López, V. Urbano-Fernández, M. Ortega-Calvo, J. Lozano-Rodríguez, I. Rivera-Benítez, M. Caballero-Valderrama, P. Iglesias-Bonilla, P. Román-Torres, Y. Corchado-Albalat, L. Mellado-Martín.

*Department of Endocrinology and Nutrition, Hospital Fundación Jimenez Díaz. Instituto de Investigaciones Biomédicas IISFJD. University Autonoma, Madrid, Spain:* A.I. de Cos, S. Gutierrez, S. Artola, A. Galdon, I. Gonzalo.

*Lipids and Vascular Risk Unit, Internal Medicine, University Hospital of Bellvitge- IDIBELL, Hospitalet de Llobregat, Barcelona, Spain:* X. Pintó, A. Galera, M. Gimenez-Gracia, E. de la Cruz, R. Figueras, M. Poch, R. Freixedas, F. Trias, I. Sarasa, M. Fanlo-Maresma, H. Lafuente, M. Liceran, A. Rodriguez-Sanchez, C. Pallarols, E. Gómez-Sanchez, V. Esteve-Luque, J. Monedero, X. Corbella, E. Corbella.

*Department of Endocrinology, IDIBAPS, Hospital Clinic, University of Barcelona, Barcelona, Spain:* A. Altés, I. Vinagre, C. Mestre, J. Viaplana, M. Serra, J. Vera, T. Freitas, E. Ortega, I. Pla, R. Olbeyra.

*Nutritional Control of the Epigenome Group. Precision Nutrition and Obesity Program, Institute IMDEA-Food, CEI UAM+CSIC, Madrid, Spain:* J.M. Ordovás, V. Micó, L. Berninches, L. Díez, M.J. Concejo, J. Muñoz, M. Adrián, Y. de la Fuente, C. Albertos, M.L. Cornejo, C. Cuesta. A. Montero., J. Aroca, B. Cáceres Sánchez, ME. Jiménez Caravera, MA. Aldavero Palacios, S. Conti Fernández, MC. Rodríguez Romero, PJ. Jiménez Pérez, V. Fernández Gutiérrez, R. Baños Morras, C. Gómez Almodóvar, P. González Escobar, AM. Ibarra Sánchez, A. Manzanares Briega M. Renata Muñoz Bieber, J. Muñoz Gutiérrez, L. Santos Larregola, C. Cassinello Espinosa, MC. Molins Santos, JM. Rodríguez Buitrago, E. Sánchez Balsalobre, C. Albertos Carrion, C. Cuesta González, L. González Torres, ME. Villahoz Loureiro, E. Arrebola Vivas, AF. Fernández Garcia, ML. Cornejo Alonso, JA. Romo Martin, L. Carabias Jaen, C. Barbero Macías, MA. Blanca De Miguel Oteo, E. Bartolomé Cobeña, MM. Adrián Sanz, MA. Angel Álvaro Sánchez, MD. Cano Pérez, MP. Lopez Morandeira, A. Montero Costa, E. Robles Fernandez, I. Alba Llacer, J. Aroca Palencia, R. Sanz Merino, MJ. Concejo Carranza, A. Garcia Romero, MC. Gómez Tabera, C. Lesmes Lora, J. Zarco Montejo, A. Campo Lopez, ME. Collado Correa, MS. Díaz Moreno, B. Doval Segura, RM. Gómez Quiroga, S. Hernando Gómez, MJ. Martínez Sanz, AM. Yunquera Alonso.

*Division of Preventive Medicine, University of Jaén, Jaén, Spain:* J.J. Gaforio, S. Moraleda, N. Liétor, J.I. Peis, T. Ureña, M. Rueda, M.I. Ballesta.

*Department of Endocrinology and Nutrition, Hospital Clínico San Carlos, Instituto de Investigación Sanitaria del Hospital Clínico San Carlos (IdISSC), Madrid, España (Spain para internacionales:* C. Moreno Lopera, C. Aragoneses Isabel, M.A. Sirur Flores, M. Ceballos de Diego, T. Bescos Cáceres, Y. Peña Cereceda, M. Martínez Abad, R. Cabrera Vélez, M. González Cerrajero, M.A. Rubio Herrera, M. Torrego Ellacuría, A. Barabash Bustelo, M. Ortiz Ramos, A. Larrad Sainz.

## Data availability statement

The original contributions presented in the study are included in the article/Supplementary material, further inquiries can be directed to the corresponding author/s.

## Ethics statement

The studies involving human participants were reviewed and approved by the protocol and procedures were implemented following the ethical standards of the Declaration of Helsinki and approved by the Institutional Ethics Review Boards of each study center. The patients/participants provided their written informed consent to participate in this study.

## Author contributions

JS-S, MM-G, MF, DC, JM, ÁA-G, JW, JeV, DR, JL-M, RE, FT, JL, LS-M, AB-C, JT, VM-S, XP, PM-M, JoV, CV, and LD designed the research. IP-G and JS-S analyzed the data and wrote the manuscript. JS-S, MM-G, MF, DC, JM, ÁA-G, JW, JeV, DR, JL-M, RE, FT, JL, LS-M, AB-C, JT, VM-S, XP, PM-M, JoV, CV, LD, SN, ER, JG-G, ET, JS, OC, AG-R, MG, FB-L, MR-C, MM, RC, EG-G, LT-S, JF-G, ZV-R, RF-C, AG, PP-O, LC-G, and HS revised the manuscript for important intellectual content and read and approved the final manuscript. IP-G, JS-S, and MM-G had full access to all of the data in the study, take responsibility for the integrity of the data and the accuracy of the data analysis. All authors contributed to the article and approved the submitted version.
