# Supplementary material for: Impact of COVID-19 pandemic on the PREDIMED-Plus randomized clinical trial: Effects on the interventions, participants follow-up, and adiposity
Source: Front Nutr. 2023 Jan 12;9:1098269. doi: 10.3389/fnut.2022.1098269 (PMC9878159; doi:10.3389/fnut.2022.1098269)
Supplement: Supplementary file 1 [file Data_Sheet_1.pdf]

Supplementary Material

- **Supplemental Material**

    - **Supplemental Material 1. Questionnaire to assess the integrity of the PREDIMED-Plus trial during and after the COVID-19 pandemic lockdown**

Supplemental Tables

- **Supplemental Table 1. Integrity of the PREDIMED-Plus intervention in the different study centers during the COVID-19 Pandemic (mean reported percentages of participants receiving the invention)**

- **Supplemental Table 2. Participants with clinically meaningful changes**

Supplemental Material 1. Questionnaire to assess the integrity of the PREDIMED-Plus trial during and after the COVID-19 pandemic

Center __ __   Date: __ __ / __ __ / __ __ __ __   **PREVENCIÓN CON DIETA MEDITERRÁNEA**

<u>Questionnaire to assess the integrity of the PREDIMED-Plus trial during and after the COVID-19 pandemic.</u>

INSTRUCTIONS: The objective of this questionnaire is to estimate the influence of the sanitary measures applied from March 2020 to June 2021. Please read each question carefully and write down the answer that best describes the situation of your center in each 3-month period.

Please specify the mean <u>PERCENTAGE</u> of lockdown/prohibition (%)the participants of your center were affected by each study period: *(<u>0%</u> = there was no lockdown in that period - <u>**100%**</u> = there was complete restriction throughout the period - Please use only the percentages <u>25%</u>, <u>50%</u> and <u>75%</u> for intermediate conditions)*

**Periods→**	**03/2020 05/2020**	**06/2020 08/2020**	**09/2020 12/2020**	**01/2021 03/2021**	**04/2021 06/2021**	**Observations**
Complete home lockdown						
Partial lockdown (county/provincial/autonomic)						
Night lockdown (curfew)						
Prohibition of going for a walk with children or pets, or playing sports outdoors						
Prohibition of attending closed places: gymnasiums / civic centers / conferences/lessons in closed rooms / places of worship / cultural activities / shopping centers.						
Prohibition of holding private meetings of ≥ 2 cohabiting persons						
Complete prohibition of going to bars/restaurants.						
Prohibition of going to bars /restaurants indoors.						

For each period, please indicate in which **<u>PERCENTAGE</u>** the different methods of **contact, data collection and intervention strategies** were used in your center: *(<u>**0%**</u>= this method was not used – <u>**100%**</u> Only used method – Please use only the percentages 25%, 50% and 75% for intermediate conditions)*

**Periods→**	**03/2020 05/2020**	**06/2020 08/2020**	**09/2020 12/2020**	**01/2021 03/2021**	**04/2021 06/2021**	**Observations**
Anthropometric measurements • measured in <u>**face-to-face**</u> interviews						
• <u>**self-reported**</u> by the participant (***not*** measured)						

Individual delivery of nutritional and physical activity recommendations: • **In-person visit**						
• by **telephone**						
• electronic media: **email, and social networks**<br>Specify the type of material used: recipes, videos, PowerPoint, etc.						
**Group** sessions • **face-to-face**						
• by **videocalls**						
**Delivery of olive oil** (in time or delayed)						
**Collection of accelerometer data**						
**Collection of blood samples**						
**Collection of fecal and urine samples**						

Supplemental Table 1. Integrity of the PREDIMED-Plus intervention in the different study centers during the COVID-19 Pandemic (mean reported percentages of participants receiving the intervention).

Center	Individual nutritional & physical activity recommendations			Group sessions		Olive oil delivery	Anthropometric data collection		Collection of blood samples	Collection of fecal/urine samples	Accelerometer data collection
	Face to face	Telephone	Electronic media	Face to face	Video calls		Face to face	Self-reported			
A	30 ± 27.4	45 ± 27.4	25 ± 0	60 ± 54.8	0 ± 0	80 ± 44.7	65 ± 48.7	35 ± 48.7	65 ± 48.7	60 ± 54.8	100 ± 0
B	20 ± 32.6	65 ± 48.7	10 ± 22.4	0 ± 0	0 ± 0	20 ± 44.7	15 ± 33.5	65 ± 48.7	0 ± 0	0 ± 0	NA
C	5 ± 11.2	70 ± 11.2	25 ± 0	5 ± 11.2	95 ± 11.2	80 ± 44.7	85 ± 33.5	15 ± 33.5	80 ± 44.7	80 ± 44.7	80 ± 44.7
D	40 ± 41.8	60 ± 41.8	45 ± 41.1	0 ± 0	100 ± 0	75 ± 0	100 ± 0	0 ± 0	40 ± 22.4	100 ± 0	60 ± 41.8
E	80 ± 44.7	20 ± 44.7	30 ± 41.1	0 ± 0	30 ± 41.1	80 ± 44.7	65 ± 48.7	20 ± 44.7	80 ± 44.7	80 ± 44.7	0 ± 0
F	60 ± 37.9	20 ± 32.6	45 ± 41.1	0 ± 0	0 ± 0	100 ± 0	60 ± 37.9	20 ± 32.6	35 ± 37.9	55 ± 37.1	55 ± 37.1
G	80 ± 44.7	20 ± 44.7	20 ± 44.7	0 ± 0	60 ± 54.8	100 ± 0	80 ± 44.7	10 ± 22.4	45 ± 32.6	80 ± 44.7	80 ± 44.7
H	40 ± 37.9	60 ± 37.9	5 ± 11.2	0 ± 0	80 ± 44.7	30 ± 20.9	40 ± 37.9	60 ± 37.9	35 ± 37.9	25 ± 25	20 ± 27.4
I	15 ± 22.4	40 ± 13.7	45 ± 11.2	15 ± 22.4	45 ± 44.7	60 ± 54.8	45 ± 32.6	55 ± 32.6	45 ± 32.6	45 ± 32.6	NA
J	5 ± 11.2	70 ± 11.2	25 ± 0	5 ± 11.2	95 ± 11.2	80 ± 44.7	85 ± 33.5	15 ± 33.5	80 ± 44.7	80 ± 44.7	60 ± 54.8
K	65 ± 22.4	30 ± 11.2	5 ± 11.2	0 ± 0	50 ± 0	75 ± 0	70 ± 11.2	30 ± 11.2	70 ± 11.2	75 ± 0	90 ± 13.7
L	65 ± 28.5	10 ± 13.7	25 ± 17.7	60 ± 37.9	40 ± 37.9	100 ± 0	80 ± 30.6	20 ± 32.6	70 ± 44.7	60 ± 37.9	5 ± 11.2
M	10 ± 22.4	20 ± 11.2	50 ± 0	10 ± 22.4	0 ± 0	70 ± 41.1	55 ± 51.2	55 ± 41.1	70 ± 41.1	75 ± 43.3	60 ± 33.5
N	50 ± 35.4	35 ± 22.4	100 ± 0	30 ± 41.1	0 ± 0	60 ± 33.5	60 ± 33.5	40 ± 33.5	60 ± 33.5	60 ± 33.5	60 ± 33.5
NY	55 ± 32.6	40 ± 33.5	25 ± 43.3	20 ± 27.4	80 ± 27.4	65 ± 41.8	55 ± 32.6	45 ± 32.6	65 ± 41.8	65 ± 41.8	65 ± 41.8
O	10 ± 13.7	65 ± 13.7	25 ± 0	0 ± 0	100 ± 0	75 ± 0	50 ± 35.4	35 ± 37.9	70 ± 41.1	65 ± 37.9	0 ± 0
P	15 ± 13.7	50 ± 17.7	75 ± 17.7	0 ± 0	35 ± 48.7	40 ± 22.4	25 ± 17.7	41 ± 20.1	55 ± 51.2	55 ± 51.2	0 ± 0
Q	60 ± 33.5	40 ± 33.5	20 ± 44.7	20 ± 32.6	0 ± 0	25 ± 35.4	75 ± 43.3	25 ± 43.3	80 ± 32.6	85 ± 33.5	65 ± 22.4
R	20 ± 44.7	20 ± 44.7	20 ± 44.7	0 ± 0	100 ± 0	40 ± 22.4	80 ± 44.7	20 ± 44.7	50 ± 30.6	50 ± 30.6	40 ± 22.4
S	65 ± 41.8	35 ± 41.8	100 ± 0	0 ± 0	0 ± 0	100 ± 0	60 ± 37.9	40 ± 37.9	80 ± 44.7	80 ± 44.7	20 ± 44.7
T	60 ± 45.4	40 ± 45.4	25 ± 35.4	0 ± 0	100 ± 0	100 ± 0	60 ± 45.4	40 ± 45.4	60 ± 45.4	55 ± 41.1	45 ± 41.1
U	65 ± 48.7	25 ± 35.4	10 ± 22.4	0 ± 0	0 ± 0	40 ± 54.8	65 ± 48.7	35 ± 48.7	30 ± 32.6	15 ± 13.7	NA
V	15 ± 13.7	35 ± 13.7	50 ± 0	0 ± 0	100 ± 0	100 ± 0	35 ± 37.9	65 ± 37.9	40 ± 54.8	40 ± 54.8	50 ± 46.8

Data are expressed as mean ± SD. Data represent the means estimated from the reported percentages during the 5 study periods (March 2020 to June 2021).
*NA: Not applicable, accelerometer data are not collected in these centers.

Supplemental Table 2. Participants with clinically meaningful changes

	No. (%) Reduction ≥5%			Increase >5%		
	Full sample	Group A	Group B	Full sample	Group A	Group B
Body weight change						
During lockdown	398 (9.5)	182 (8.9)	216 (10.0)	301 (7.2)	133 (6.5)	168 (7.8)
Post lockdown	534 (13.0)	249 (12.5)	285 (13.5)	419 (10.0)	179 (8.7)	240 (11.1)
Body mass index						
During lockdown	380 (9.7)	176 (9.3)	204 (10.1)	333 (8.5)	153 (8.0)	180 (8.9)
Post lockdown	523 (13.4)	246 (13.0)	277 (13.8)	463 (11.8)	198 (10.4)	265 (13.2)
Waist circumference						
During lockdown	198 (6.4)	80 (5.3)	118 (7.4)	331 (10.7)	146 (9.6)	185 (11.6)
Post lockdown	278 (8.9)	114 (7.5)	164 (10.2)	428 (13.7)	199 (13.1)	229 (14.3)
